# Nutritional status of tribal and non-tribal school-going children in rural Bangladesh: A comparative study

**DOI:** 10.1186/s12889-024-20487-9

**Published:** 2024-10-28

**Authors:** Reazul Karim, Ramendra Nath Kundu, Sifat Hossain, Susmita Bharati, Premananda Bharati, Golam Hossain

**Affiliations:** 1 Health Research Group, Department of Statistics, University of Rajshahi, Rajshahi, 6205 Bangladesh; 2 Department of Anthropology, UGC-NET), West Bengal State University, West Bengal, India; 3 Sociological Research Unit, Indian Statistical Institute, Kolkata, West Bengal India; 4 Former Professor and Head, Biological Anthropology Unit, Indian Statistical Institute, Kolkata, West Bengal India

**Keywords:** School-going children, Nutritional status, Tribal and non-tribal, Rural area, Bangladesh

## Abstract

**Background:**

Inadequate nutrition of school-going children is a major concern in Bangladesh, and it can negatively affect their productivity. It is important to consider the food pattern, socio-cultural, and economic differences between tribal (T) and non-tribal (NT) communities in Bangladesh when evaluating their nutritional status. This study aimed to investigate the nutritional status of school-going children in the rural area of Rajshahi district’s High Barind Tract (HBT) region of Bangladesh. Additionally, we compared the nutritional status between T and NT school-going children in the same area.

**Methods:**

This was a cross sectional household study. Data were collected from T and NT households in the HBT region in the Rajshahi district of Bangladesh, from January to June of 2019. A total of 500 (T 81, NT 419) school-going children aged 6–13 years were selected as samples using mixed sampling, including convenience sampling (non-probability) and simple random sampling (probability) methods. Nutritional status was assessed using body mass index-for-age z-score (BAZ) and height-for-age z-score (HAZ) according to WHO guidelines. Thinness was defined as BAZ < -2SD and stunting as HAZ < -2SD. Descriptive statistics, Z-proportional test, and logistic regression model were used to analyze the effect of selected independent variables on nutritional status of T and NT children.

**Results:**

Among school-going children, 15.20% were suffering from thinness (T 12.30% and NT 15.80%) and 17.80% stunting (T 13.60% and NT 18.60%), respectively. The difference in thinness (*p* > 0.05) and stunting (*p* > 0.05) were not significant between T and NT. The distribution of BAZ and HAZ of T and NT children were normally distributed, and were positioned negatively compared to the WHO standards. The logistic model identified the following factors for thinness: (i) mother with non-or-primary education (aOR = 1.89, 95% CI: 1.05–3.43, *p* < 0.05), (ii) underweight mother (aOR = 3.86, 95% CI: 1.48–10.06, *p* < 0.01), and (iii) underweight father (aOR = 4.12, 95% CI: 1.50-11.29, *p* < 0.01). For stunting, the factors were: (i) mother as a housewife (aOR = 2.79, 95% CI: 1.16–6.71, *p* < 0.05), (ii) father working as labour (aOR = 1.77, 95% CI: 1.01–3.278, *p* < 0.05), (iii) severe food insecurity in the household (aOR = 2.37, 95% CI: 1.23–4.54, *p* < 0.05), and (iv) children playing outside regularly more than 2 h (aOR = 2.19, 95% CI: 1.31–3.67, *p* < 0.01).

**Conclusion:**

In rural Bangladesh, the nutritional status of T and NT school-going children did not show significant defferences. However, the mean z-score values for both groups of children were lower than the WHO standard, indicating that both communities have poor nutritional status.

## Introduction

Undernutrition is a serious public health issue that adversely affects children’s ability to learn and can lead to lower academic performance [1, 2]. School age is a pivotal period in the lives of children when important physical, mental, emotional, and social transformations occur [3]. This period includes the beginning of puberty, growth spurts, and changes in the brain’s influence on a young person’s emotions, behaviour regulation, and decision-making [4]. When children suffer from nutritional deficiencies, they become more susceptible to infections and may suffer negative effects on their mental development [5]. A balanced diet is crucial for maintaining endurance, improving physical and cognitive development, and enhancing efficiency [6].

Malnutrition involves a deficiency, excess, or imbalance of energy and other essential nutrients, leading to changes in body composition, function, and health [7]. It encompasses all forms of poor nutrition, including severe hunger and undernutrition as well as obesity [7]. Over 200 million schoolchildren worldwide are affected by malnutrition, predominantly undernutrition [8]. Globally, around a third of children are not growing properly due to lack of nutrition, like stunting, wasting, and overweight. About one in three schoolchildren are consuming the minimum recommended number of food groups, with only 20% of children from low-income rural families meeting this guideline. According to UNICEF and WHO, schoolchildren should consume a minimum of five out of the eight food groups [9].

Malnutrition is a major concern for global health, affecting both infants and young adolescents [10]. Problems related to both undernutrition and overnutrition have been identified in studies focusing on school-aged children in LMICs like Nigeria and Mexico, including issues with food insecurity, dietary variety, and patterns [11, 12]. As part of the Digital Bangladesh Vision, the government aims to digitize its human resources and development processes. Therefore, it is essential to have a specific plan addressing child malnutrition. The government of Bangladesh has established targets aligned with the Sustainable Development Goals (SDGs) to improve nutrition and health by 2030 [13]. However, due to very limited research focusing on primary schoolchildren (ages 5–9 years) and early adolescents (ages 10–14 years), especially in tribal communities in Bangladesh, limits our understanding of the prevalence of malnutrition in these age groups and hampers the development of effective strategies [14].

The Rajshahi district, located in the north-western region of Bangladesh, is known for its unique geography and ethnic diversity. The district is mainly made up of the Barind tract, home to various tribal communities [15]. These tribes have distinct food habits, habitations, socio-cultural practices, and economic activities that set them apart from non-tribals.

Some studies in Bangladesh have highlighted malnutrition and poor food security outcomes among tribal children, emphasizing the need to address these issues and enhance the overall health and well-being of this marginalized population [16, 17]. There is a lack of research comparing the nutritional status of school-going tribal and non-tribal children in the Rajshahi district. Therefore, it is crucial to conduct research on nutritional status and to examine how socioeconomic and other contextual factors influence children’s nutrition.

The study aims to investigate the nutritional status of school-going children in rural HBT region of Rajshahi district, Bangladesh. Additionally, a comparison of the nutritional status between tribal and non-tribal children was conducted.

## Methods

### Study design and target population

An extensive study was conducted to evaluate the nutritional status of school-going children, aged 6 to 13, and living in rural areas of the HBT in Rajshahi District, Bangladesh. The study employed a cross-sectional design and classified the participants into two groups: tribal children (T) and non-tribal children (NT) to compare their nutritional status. The survey included students from primary and junior high schools, in grades 1 to 7. The Orao, Santal, and Rajvanshi tribal groups were surveyed as they were present in the region. Furthermore, the study examined the nutritional status of parents to understand its impact on their children’s well-being. Data was collected through a household survey from January to June 2019.

### Study area

The study focused on the HBT areas, primarily covering the districts of Rajshahi [18, 19]. This region stands out from other parts of Bangladesh due to its terraced landscape, low-fertility soil, limited vegetation, lack of major river channels, and an unpredictable and comparatively low rainfall pattern, with a prolonged dry spell from October to May. The HBT is situated between 9 and 47 m above sea level and does not experience annual flooding, unlike the floodplain regions of Bangladesh [20]. It is also characterized by low literacy rates and high poverty levels, with 2.3% of the population being ethnic minorities and among the most underprivileged groups [15].

### Sample selection procedures

**Fig. 1:**
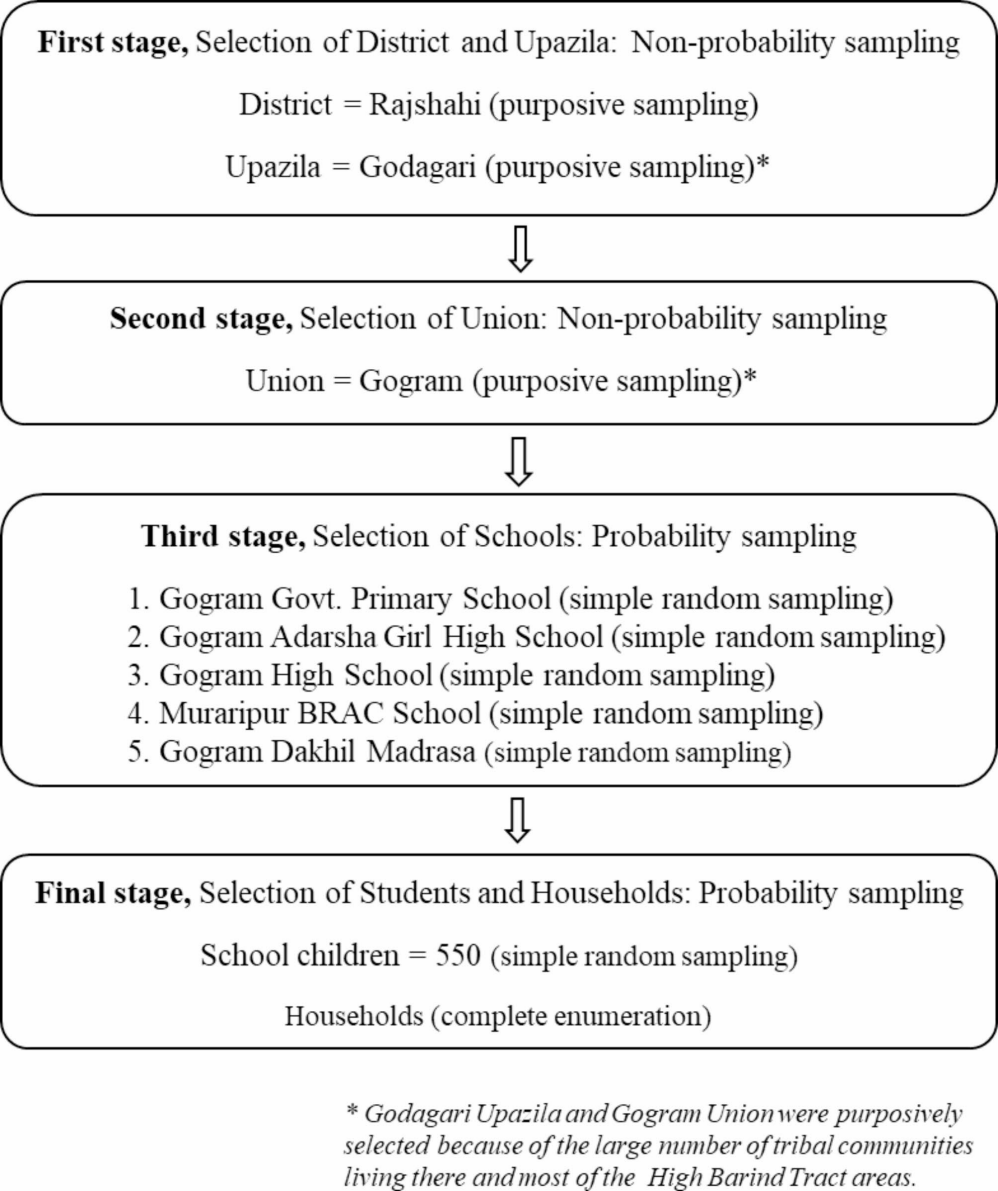
Sample selection procedure

In this study, we used a multistage mixed sampling technique (combining both probability and non-probability sampling) to select our primary sample from the population, with the primary sampling unit being schools. From the Rajshahi district, Godagari upazila were selected; from the selected upazila, the Gogram union selected using purposive sampling as well, due to its relatively high number of tribal communities. We then used simple random to select five schools under this union. Once our primary sample was established, we reached out to school authorities and surveyed households with children attending our selected schools through complete enumeration. The participants in the study were divided into two categories: T children and NT children, based on their attendance at the selected schools. In this study, we selected one child from each household. If there were multiple children in a household, one child was chosen via lottery. Before the data collection, the field employees underwent thorough training to ensure they were well-equipped. The detailed sample selection procedure is outlined in Fig. 1.

### Inclusion criteria

We included children in our study who were living with their parents, in the HBT areas of Rajshahi district, attending Grade 1 to 7 classes, aged 6 to 13, and not suffering from severe illness.

### Outcome variable

The nutritional status of school-going children was the outcome variable of the study. Based on WHO guidelines, the nutritional status of school-going children was measured by the z-score of body mass index (BMI)-for-age (BAZ) and height-for-age (HAZ). The BAZ was classified as thinness (BAZ < − 2 SD) and (ii) non-thinness (BAZ ≥ − 2 SD), while the HAZ was classified as stunting (HAZ < − 2 SD) and non-stunting (HAZ ≥ − 2 SD).

### Independent variables

The study included socioeconomic, demographic, health and nutritional factors as independent variables, many of which were selected from previous research [21, 22]. All independent variables are mentioned in Table 1.

### Statistical analysis

The distribution and prevalence of nutritional status were determined using descriptive statistics, which included mean and standard deviation (SD) for continuous variables, while frequencies and percentages for discrete variables. Inferential statistics, including mean difference (MD) with Confidence interval (CI), z-proportion test, and logistic regression were employed to determine inter-community differences and factors influencing the nutritional status of schoolchildren.

The MD test was used to compare age, height, weight, BAZ and HAZ between T and NT children. The Kolmogorov-Smirnov test was used to test the normality of the data. The test confirmed that our data were normally distributed and met MD’s standard assumption. The z-proportion test was utilized to find the difference in the prevalence (proportion) of nutritional status between T and NT children. The z-proportional test is a statistical method that is employed to identify the significant variations in the proportion between two groups. The test works by calculating a z-score, which is a measure of the deviation of the observed difference in proportions from the expected difference under the null hypothesis. The logistic regression model was used to examine the effect of independent variables on dependent variable. The results of the multiple logistic regression model were presented by adjusted odds ratio (aOR) with 95% confidence interval (CI) for the effects of the associated factors. The multicollinearity among independent variables was examined using a Variance Inflation Factor (VIF); if 0 < VIF < 5, there is no evidence of a multicollinearity problem. We did not find multicollinearity problems among independent variables in the multiple logistic regression model. To calculate BAZ and HAZ scores, we used the WHO AnthroPlus software, as it’s suitable for children aged 5 to 19 years. All statistical analyses were performed using the Statistical Package for the Social Sciences (SPSS, version 23.0), and we considered results statistically significant at *p* < 0.05.

## Results

### Distribution of sociodemographic and children’s physical activities factors

Out of a total of 500 children in the sample, 48.40% were boys and 51.60% were girls. Among the boys, 15% belonged to the T ethnicity while the remaining 83.80% were NT. For the girls, 17.05% were from the T and the rest (82.95%) were NT. About 57.20% of mothers had completed secondary and higher education, and 36.40% had completed primary education. Among the mothers with secondary and higher education, 58.48% were NT, while 50.61% of the tribal mothers had the same level of education. Looking at the fathers, 44% had completed primary education, and 38.26% had completed secondary and higher education. Comparatively, 50.00% of the fathers with primary education were T, while 42.68% were NT. The data also showed that the majority of mothers (85.60%) were housewives, with 28.39% being T and 96.66% being NT. It was noted that 14.40% of mothers worked as daily labourers, among them 71.61% came from T. In terms of the fathers’ occupations, almost 73% worked as daily labourers; among them, 85.39% were T and 69.76% were NT. More than half (50.80%) of households had 1–4 members among them 43.21% being T and 52.27% being NT. Over one-third (34.60%) of households had an average monthly income in the range of 7,501 − 10,000 BDT, out of which 40.74% were T and 33.41% were NT. The data shows that almost half of the households experienced food insecurity, with 36% facing moderate insecurity and 11.20% facing severe insecurity. Most of the mothers were of normal weight (63.80%), with a higher proportion were T (69.14%) than NT (62.77%). 25.80% of mothers were overweight and obese, with a high proportion were NT (28.16%) than T (13.58%). Data also revealed that nearly 70% fathers had a normal nutritional status, among them 86.84% were T and 65.85% were NT. 22.63% fathers were overweight or obese, with a higher proportion were NT (25.86%) than T (5.27%). Around 60% of households did not have access to a hygienic toilet, with a higher proportion (62.96%) in T households compared to NT households (58.48%). More than 50% of children spent over two hours daily playing outdoor games, with 58.03% belonging to T and 50.59% NT communities.

Moreover, 82.80% of schoolchildren watched TV for less than two hours regularly, with 86.42% belonging to T and 82.11% NT communities (Table 1).

**Table 1:** Distribution of the children and their parents’ socioeconomic and demographic factors (father: *n* = 486; mother: *n* = 500)

	Name of Variables	Study participants		
		Tribal (n, %)	Non-tribal (n, %)	Total (n, %)
Child gender				
	Boy	37 (15.29)	205 (84.71)	242 (48.40)
	Girl	44 (17.05)	214 (82.95)	258 (51.60)
Mother’s education				
	Non-educated	8 (9.88)	24 (5.73)	32 (6.40)
	Primary	32 (39.51)	150 (35.79)	182 (36.40)
	Secondary and higher	41 (50.61)	245 (58.48)	286 (57.20)
Father’s education				
	Non-educated	11 (14.47)	76 (18.54)	87 (17.92)
	Primary	38 (50.00)	175 (42.68)	213 (43.82)
	Secondary and higher	27 (35.53)	159 (38.78)	186 (38.26)
Mother’s occupation				
	Housewife	23 (28.39)	405 (96.66)	428 (85.60)
	Labourer	58 (71.61)	14 (3.35)	72 (14.40)
Father’s occupation				
	Labourer	65 (85.39)	286 (69.76)	351 (72.22)
	Farmers & others	11 (14.61)	124 (30.24)	135 (27.78)
Total household members				
	≤ 4	35 (43.21)	219 (52.27)	254 (50.80)
	> 4	46 (56.79)	200 (47.73)	246 (49.20)
Household average monthly income				
	≤ 7500 BDT	12 (14.81)	55 (13.13)	67 (13.40)
	7501-10,000 BDT	33 (40.74)	140 (33.41)	173 (34.60)
	> 10,000 BDT	36 (44.45)	224 (53.45)	260 (52.00)
Household food security status				
	Food secured	30 (37.03)	234 (55.84)	264 (52.80)
	Moderate insecurity	39 (48.15)	141 (33.65)	180 (36.00)
	Severe insecurity	12 (14.82)	44 (10.51)	56 (11.20)
Mother’s BMI				
	Underweight	14 (17.28)	38 (9.07)	52 (10.40)
	Normal weight	56 (69.14)	263 (62.77)	319 (63.80)
	Overweight & obese	11 (13.58)	118 (28.16)	129 (25.80)
Father’s BMI				
	Underweight	6 (7.89)	34 (8.29)	40 (8.23)
	Normal weight	66 (86.84)	270 (65.85)	336 (69.14)
	Overweight & obese	4 (5.27)	106 (25.86)	110 (22.63)
Households having hygienic toilet				
	Yes	30 (37.04)	174 (41.52)	204 (40.80)
	No	51 (62.96)	245 (58.48)	296 (59.20)
Children regularly play outside				
	≤ 2 h	34 (41.97)	207 (49.41)	241 (48.20)
	> 2 h	47 (58.03)	212 (50.59)	259 (51.80)
Children watch TV regularly				
	≤ 2 h	70 (86.42)	344 (82.11)	414 (82.80)
	> 2 h	11 (13.58)	75 (17.89)	86 (17.20)

The research revealed that T children had a slightly higher mean age (9.35 ± 2.20 years) compared to NT children (9.18 ± 2.05 years). The mean height of T children was slightly greater (129.16 ± 13.86 cm) than that of NT children (128.93 ± 14.21 cm). T children also had a slightly lower mean weight (26.70 ± 8.30 kg) in comparison to NT children (27.79 ± 8.79 kg). The mean BAZ of NT children was higher (0.16 ± 0.37) than that of T children (0.12 ± 0.33). Moreover, the mean HAZ of NT children was higher (0.19 ± 0.39) compared to T children (0.14 ± 0.35). However, the result found that the mean differences of all these variables, such as age, anthropometric measurements, and nutritional indicators, did not show statistically significant difference (*p* > 0.05) between T and NT children (Table 2).

**Table 2:** Distribution of demographic and anthropometric characteristics of school-going children by ethnicity

Variables	Tribal (*n* = 81)	Non-tribal (*n* = 419)	MD	95% CI for MD		*p*-value	Total (*n* = 500)
	Mean ± SD	Mean ± SD		Lower	Upper		Mean ± SD
Age (year)	9.35 ± 2.20	9.18 ± 2.05	0.17	−0.32	0.66	0.567	9.21 ± 2.08
Height (cm)	129.16 ± 13.86	128.93 ± 14.21	0.23	−3.14	3.60	0.459	128.97 ± 14.14
Weight (kg)	26.70 ± 8.30	26.79 ± 8.79	−0.08	−2.16	1.98	0.570	26.77 ± 8.70
BAZ	0.12 ± 0.33	0.16 ± 0.37	−0.03	−0.12	0.05	0.345	0.15 ± 0.36
HAZ	0.14 ± 0.35	0.19 ± 0.39	−0.50	−0.14	0.04	0.246	0.18 ± 0.38

Note: SD, Standard deviation; MD, Mean difference; CI, Confidence interval

### Comparison of undernutrition prevalence between tribal and non-tribal schoolchildren

It was revealed that the prevalence of thinness and stunting of school-going children were 15.20% (T 12.30% and NT 15.80%) and 17.80% (T 13.60% and NT 18.60%). Z-proportion test demonstrated that the difference in thinness and stunting between T and NT children were not statistically significant (*p* > 0.05). It was found that the thinness of T and NT school boys was 13.51% and 15.12%, respectively; while among girls it was 11.36% and 16.35% respectively. On the other hand, the stunting of T and NT school boys was 21.62% and 18.05%, respectively; while among the girls it was 6.81% and 19.16%, respectively. The Z-proportion test showed that there were no statistically significant differences in the proportions of thinness and stunting between T and NT children, including boys and girls separately (*p* > 0.05) (Table 3).

**Table 3:** Comparison of nutritional status between tribal and non-tribal schoolchildren

Nutritional status		Tribal (*n*, %)	Non-tribal (*n*, %)	Proportional z- value	*p*-value
BMI-for-age					
	***Boy***				
	Thinness	5 (13.51)	31 (15.12)	−0.093	0.928
	Non-thinness	32 (86.49)	174 (84.88)	0.233	0.818
	***Girl***				
	Thinness	5 (11.36)	35 (16.35)	−0.287	0.771
	Non-thinness	39 (88.64)	179 (83.65)	0.781	0.435
Total	Thinness (15.20%)	81( 12.30)	419(15.80)	−0.802	0.424
Height-for-age					
	***Boy***				
	Stunted	8 (21.62)	37 (18.05)	0.237	0.810
	Non-stunted	29 (78.38)	168 (81.95)	−0.459	0.645
	***Girl***				
	Stunted	3 (6.81)	41 (19.16)	1.963	0.055
	Non-stunted	41 (93.19)	173 (80.84)	1.938	0.052
Total	Stunted (17.80%)	81(13.60)	419(18.60)	−1.077	0.280

**N.B.**: The result is significant at *p* < 0.05. Proportional z-value derived from a z-proportional test between tribal and non-tribal sample distribution.

### Comparison in BAZ and HAZ of tribal and non-tribal children with WHO reference

**Fig. 2:**
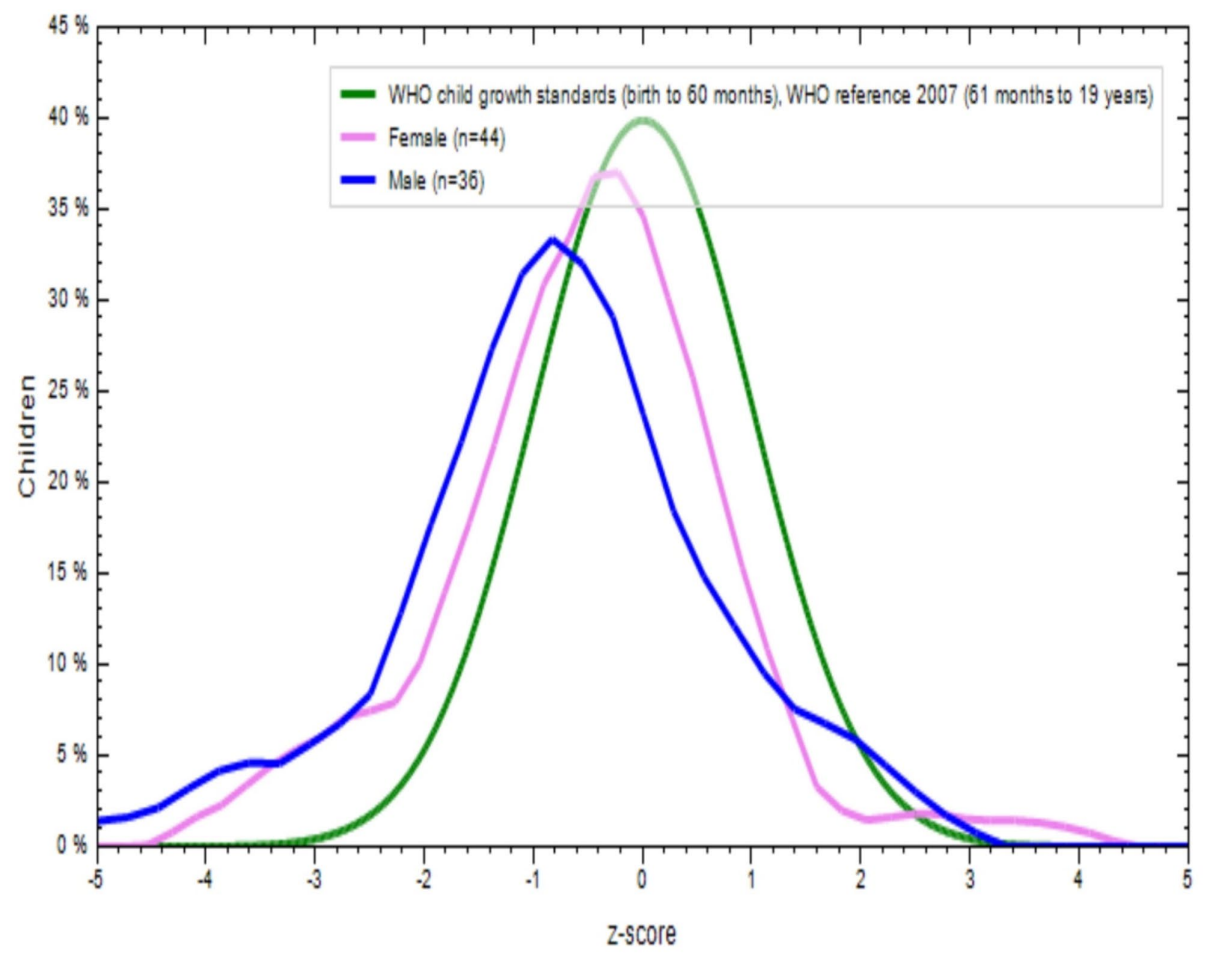
Distribution of BMI for age Z-score (thinness) of tribal school children

**Fig. 3:**
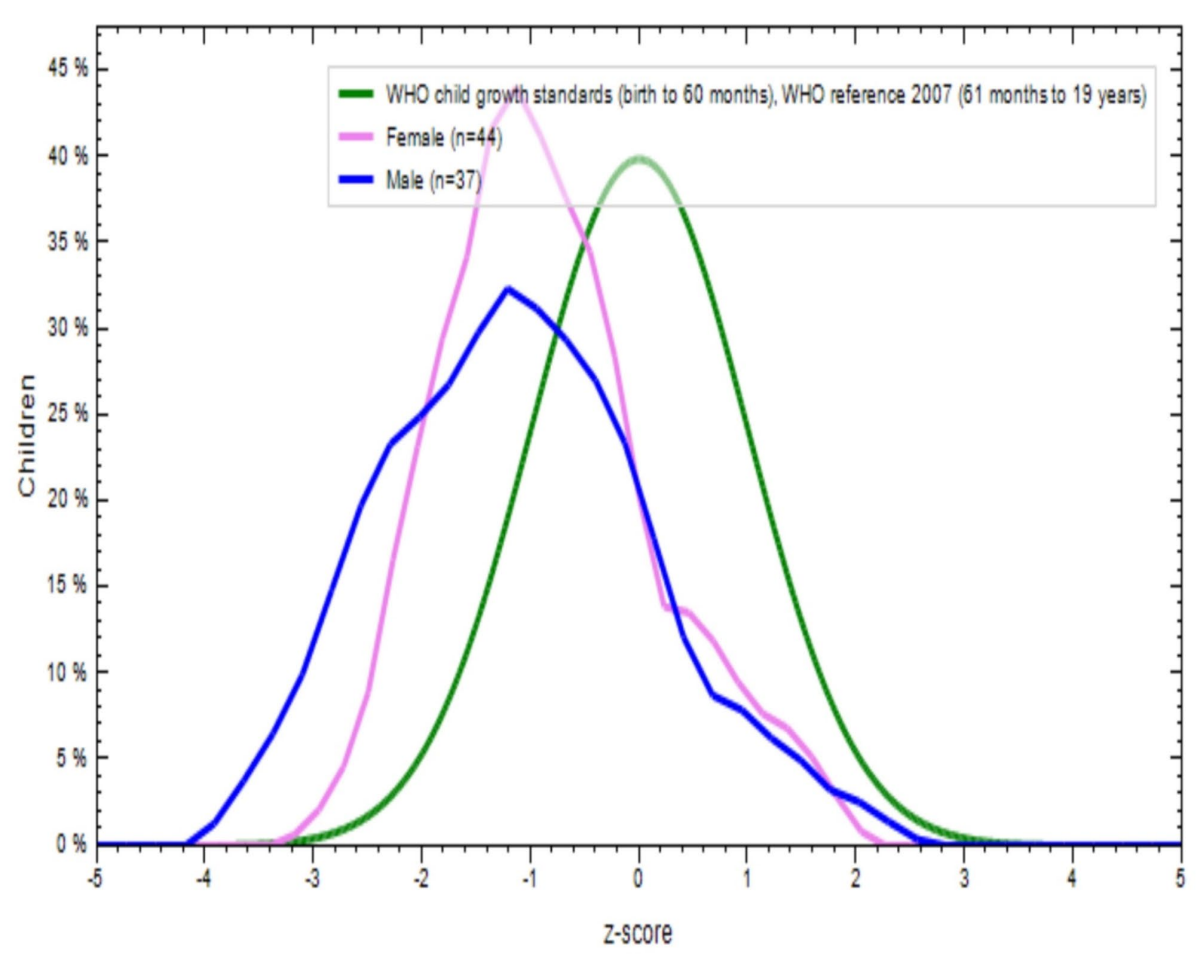
Distribution of height for age Z-score (stunting) of tribal school children

**Fig. 4:**
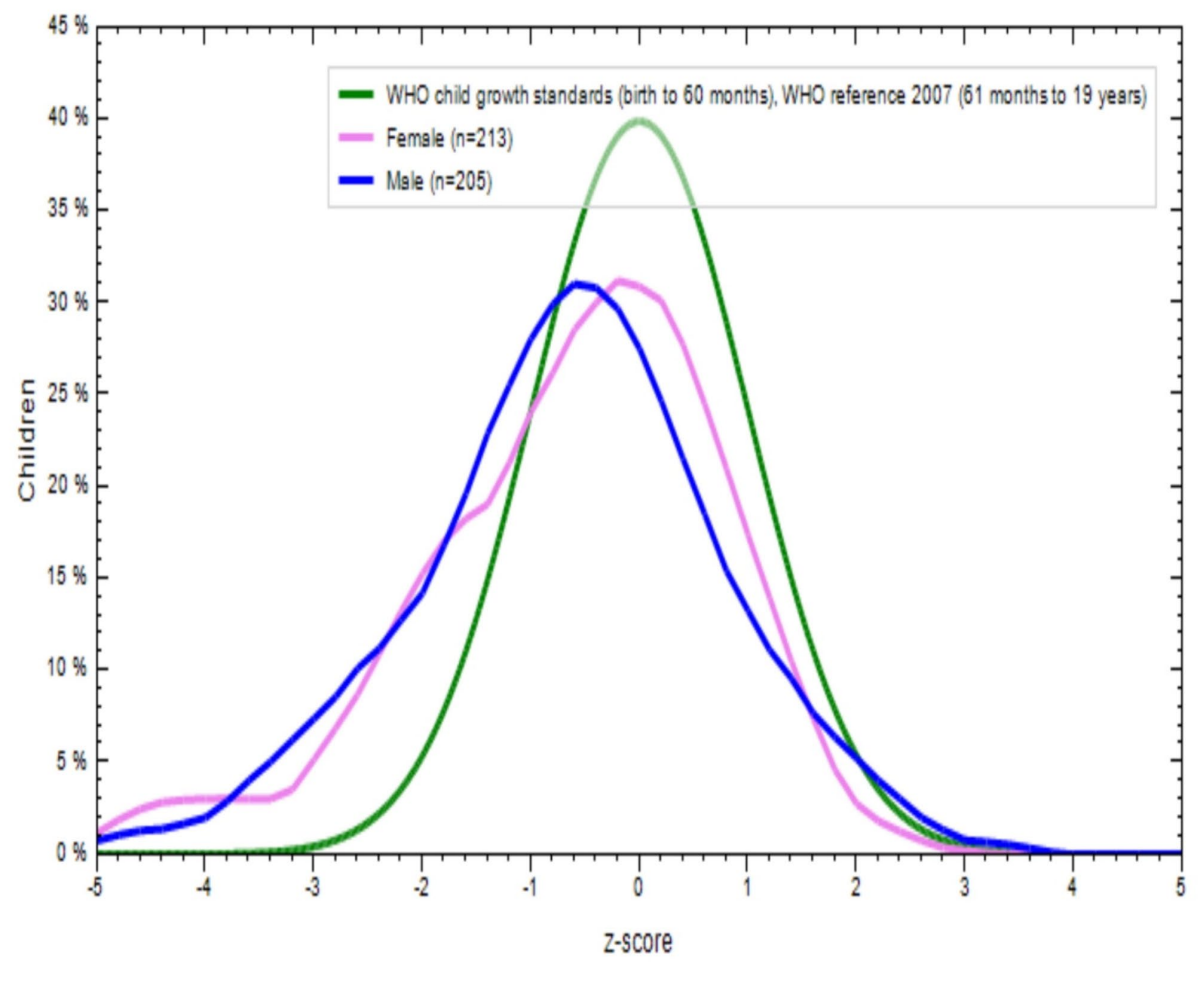
Distribution of BMI for age Z-score (thinness) of non-tribal school children

**Fig. 5:**
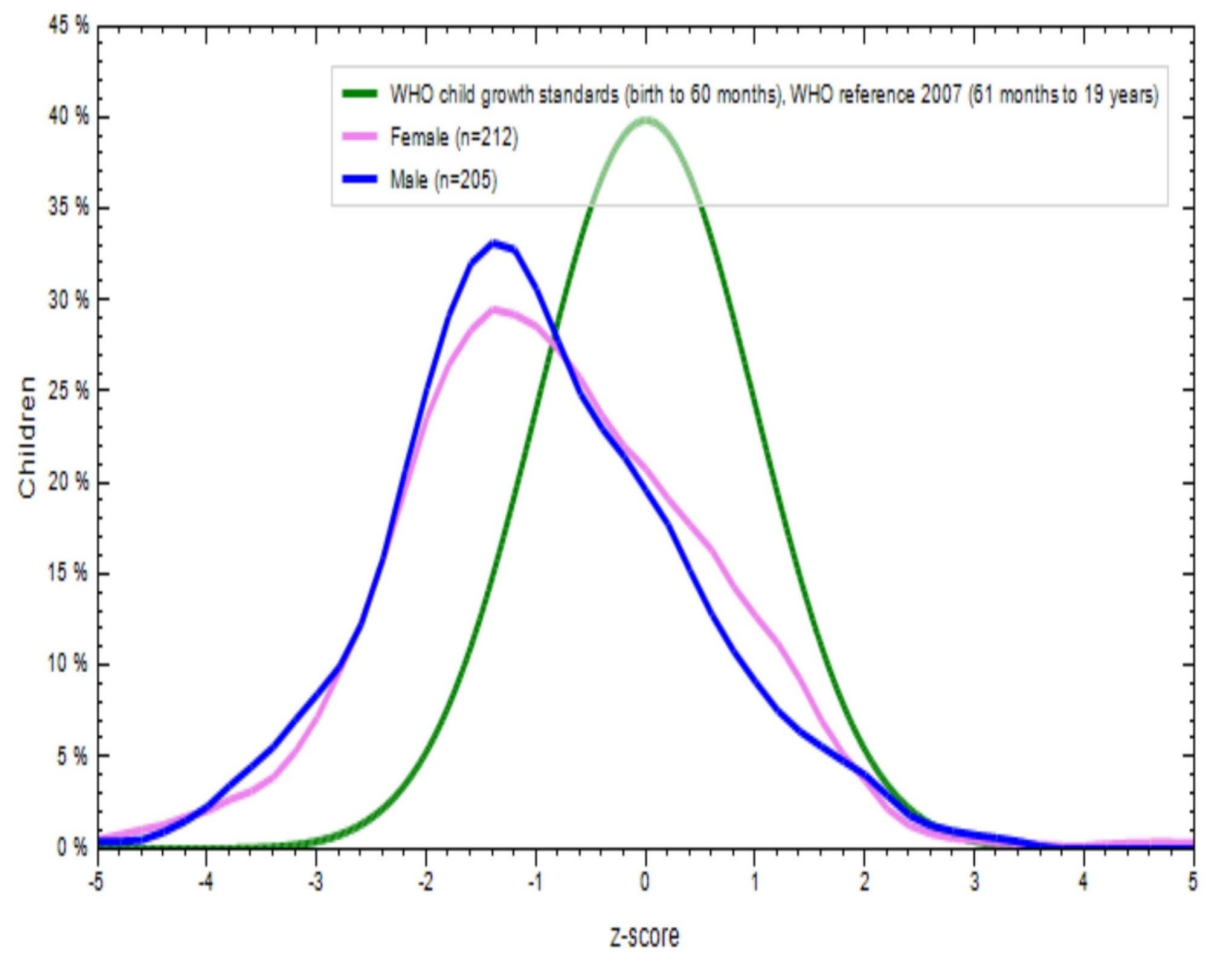
Distribution of height for age Z-score (stunting) of non-tribal school children

Figures 2, 3, 4 and 5 show a comparison of BMI-for-age (BAZ) and height-for-age (HAZ) z-scores for boys (blue line) and girls (violet line) from both school-going T and NT children. These scores were compared with the WHO standards z-scores (green line). The BAZ and HAZ z-scores for T children from both sexes were more or less normally distributed, but the mean values for both indicators were lower than the WHO standards (Figs. 2 and 3). Similar results were found for NT children for both nutritional status indicators (Figs. 4 and 5). This indicates that both T and NT children had poor nutritional status in terms of BAZ and HAZ, as their curves were located below the WHO reference curve.

### Effect of socio-economic factors on nutritional status among school-going children

The logistic regression model demonstrated that children of non or primary educated mother’s had a 1.89 times the odds of having thinness compared to children whose mother were secondary or higher educated (aOR = 1.89, 95% CI: 1.05–3.43, *p* < 0.05). The children of underweight mother’s had a 3.86 times the odds of having thinness compared to overweight mother’s children (aOR = 3.86, 95% CI: 1.48–10.06, *p* < 0.01). It was also found that children whose fathers were underweight were more likely to be thin than children whose fathers were overweight (aOR = 4.12, 95% CI: 1.50-11.29, *p* < 0.01) (Table 4). The goodness of fit of the multiple logistic model was checked by Omnibus and Hosmer and Lemeshow test, and the tests provided that our selected model was good fitted (Omnibus test (Chi-square value = 30.004; *p* = 0.026) and Hosmer and Lemeshow test (Chi-square value = 10.001; *p* = 0.265).

**Table 4:** Effect of socio-economic, demographic health related variables on thinness of tribal and non-tribal school-going children

Independent variables and groups		cOR (95% CI), *p*-value	aOR (95% CI), *p*-value
Ethnicity			
	Non-tribal	1 (reference)	
	Tribal	0.75 (0.37–1.54), 0.436	
Child gender			
	Girl	1 (reference)	
	Boy	0.95 (0.58–1.55), 0.845	
Mother’s education			
	Secondary & higher	1 (reference)	1 (reference)
	Non & Primary	1.81(1.11–2.96), 0.018	1.89 (1.05–3.43), 0.035
Father’s education			
	Secondary & higher	1 (reference)	
	Non & primary	1.13 (0.54–2.40), 0.742	
Mother’s occupation			
	Labourer	1 (reference)	
	Housewife	1.13 (0.55–2.32), 0.738	
Father’s occupation			
	Farmers & others	1 (reference)	
	Labourer	1.23 (0.70–2.19), 0.472	
Total household members			
	> 4 members	1 (reference)	
	≤ 4 members	1.03 (0.63–1.67), 0.922	
Household average monthly income			
	> 10,000 BDT	1 (reference)	1 (reference)
	≤ 7500 BDT	0.54 (0.22–1.34), 0.183	0.43 (0.16–1.16), 0.096
	7501-10,000 BDT	1.15 (0.69–1.94), 0.588	
Household food security status			
	Food secured	1 (reference)	
	Moderate insecurity	1.40 (0.60–3.29), 0.441	
	Severe insecurity	1.13 (0.46–2.77), 0.791	
Mother’s BMI			
	Overweight & obese	1 (reference)	1 (reference)
	Underweight	5.29 (2.21–12.67), 0.001	3.86 (1.48–10.06), 0.005
	Normal weight	2.21 (1.09–4.51), 0.029	1.78 (0.84–3.74), 0.132
Father’s BMI			
	Overweight & obese	1 (reference)	1 (reference)
	Underweight	6.04 (2.36–15.50), 0.001	4.12 (1.50-11.29), 0.006
	Normal weight	2.01 (0.95–4.23), 0.066	1.65 (0.76–3.59), 0.209
Households having hygienic toilet			
	No	1 (reference)	
	Yes	0.72 (0.43–1.20), 0.206	
Children regularly play outside			
	> 2 h	1 (reference)	
	≤ 2 h	1.16 (0.71–1.89), 0.555	
Children watch TV regularly			
	> 2 h	1 (reference)	
	≤ 2 h	0.82 (0.44–1.52), 0.525	

**N.B.**: cOR = Crude odds ratio; aOR = Adjusted odds ratio; CI = Confidence interval; 1 (reference) = Reference category, Education = Formal institutional education.

It was found that children of housewife mothers were more likely to have stunting compared to non-housewife (labourer) mothers (aOR = 2.79, 95% CI: 1.16–6.71, *p* < 0.05). Stunting was 1.77 times more likely to occur in children of labourer fathers (aOR = 1.77, 95% CI: 1.01–3.28, *p* < 0.05) than in non-labor fathers (farmers & others). Children living in a household having severe food insecurity were more likely to suffer from stunting (aOR = 2.37, 95% CI: 1.23–4.54, *p* < 0.05) compared to children living in a food security household. Children who played outside for more than 2 h per day were more likely to have stunting (aOR = 2.19, 95% CI: 1.31–3.67, *p* < 0.01) than children who played for less or equal 2 h per day (Table 5). Omnibus test (Chi-square value = 40.371; *p* = 0.027) and Hosmer and Lemeshow test (Chi-square value = 5.402; *p* = 0.714) showed that our selected model was good fitted (Table 5).

**Table 5:** Effect of socio-economic, demographic health related variables on stunting of tribal and non-tribal school-going children

Independent variables and groups		cOR (95% CI), *p*-value	aOR (95% CI), *p*-value
Ethnicity			
	Non-tribal	1 (reference)	
	Tribal	0.69 (0.35–1.36), 0.280	
Child gender			
	Girl	1 (reference)	
	Boy	1.11 (0.70–1.76), 0.653	
Mother’s education			
	Secondary & higher	1 (reference)	
	Non & Primary	0.81 (0.30–2.21), 0.687	
Father’s education			
	Secondary & higher	1 (reference)	1 (reference)
	Non & primary	1.55 (0.82–2.93), 0.174	0.83 (0.39–1.76), 0.629
Mother’s occupation			
	Labourer	1 (reference)	1 (reference)
	Housewife	2.65 (1.11–6.31), 0.028	2.79 (1.16–6.71), 0.022
Father’s occupation			
	Farmers & others	1 (reference)	1 (reference)
	Labourer	1.89 (1.05–3.38), 0.033	1.77 (1.01–3.28), 0.045
Total household members			
	> 4 members	1 (reference)	
	≤ 4 members	1.04 (0.66–1.65), 0.854	
Household average monthly income			
	> 10,000 BDT	1 (reference)	
	≤ 7500 BDT	1.25 (0.63–2.49), 0.527	
	7501-10,000 BDT	1.27 (0.77–2.09), 0.349	
Household food security status			
	Food secured	1 (reference)	1 (reference)
	Moderate insecurity	0.88 (0.52–1.49), 0.638	0.89 (0.53–1.51), 0.668
	Severe insecurity	2.37 (1.24–4.53), 0.009	2.37 (1.23–4.54), 0.015
Mother’s BMI			
	Overweight & obese	1 (reference)	
	Underweight	1.04 (0.46–2.37), 0.922	
	Normal weight	0.91 (0.54–4.155), 0.732	
Father’s BMI			
	Overweight & obese	1 (reference)	
	Underweight	0.50 (0.16–1.57), 0.234	
	Normal weight	1.04 (0.60–1.81), 0.894	
Household having hygienic toilet			
	No	1 (reference)	
	Yes	0.78 (0.49–1.26), 0.306	
Children regularly play outside			
	> 2 h	1 (reference)	1 (reference)
	≤ 2 h	2.20 (1.36–3.58), 0.001	2.19 (1.31–3.67), 0.003
Children watch TV regularly			
	> 2 h	1 (reference)	
	≤ 2 h	1.28 (0.72–2.28), 0.405	

**N.B.**: cOR = Crude odds ratio; aOR = Adjusted odds ratio; CI = Confidence interval; 1 (reference) = Reference category, Education = Formal institutional education.

## Discussion

The purpose of this study was to investigate the nutritional condition of school-going children in rural HBT areas in northern Bangladesh. We attempted to determine the difference in nutritional status between T and NT children living in the same location, and our selected tools/models yielded some notable results, which are described separately below.

The study revealed that thinness and stunting were less prevalent among T children than in NT children. A study conducted in Sherpur district, Bangladesh found that 15.1% of Garo adolescents suffered from stunting [23]. This percentage is quite close to the results of the present study, which shows a stunting prevalence of 13.6%. Another study on tribal children aged 6–23 months in the Chittagong Hill Tracts of Bangladesh revealed that consuming animal-source foods has a positive association with HAZ (beta = 0.073; 95% CI: 0.051–0.096), indicating an improvement in nutritional status [17]. In the present study, it was also found that food insecurity significantly increases the prevalence of stunting. These findings highlight the importance of animal-source foods in improving the nutritional status of children and the need to address food insecurity to prevent stunting. A similar finding was reported in northeastern India, where a comparative study of school-going Chakma tribal and non-tribal Bengali girls revealed that non-tribal had a greater prevalence of undernutrition than tribal, with thinness T = 3.70% vs. NT = 11.29% and stunting T = 15.64% vs. NT = 16.14%, respectively [24]. The prevalence of thinness and stunting among Bangladeshi school-going children was lower than in some other low- and middle-income countries (LMICs), such as Northwest Ethiopia (stunting: 25.5%), Sudan (thinness: 32.3% and stunting: 22.1%), Pakistan’s Faisalabad (stunting: 45.8%) and Karachi (stunting: 26.3%) [25–28]; and higher than South Tongu of Ghana (thinness: 12.1% and stunting: 10.4%), Vietnam (thinness: 5.4% and stunting: 14.0%), Ebonyi of Nigeria (thinness: 7.2% and stunting: 9.9%), Denkyembour of eastern Ghana (thinness: 6.7% and stunting: 16.7%) [29–32]. Hence, among LMICs, school-going children in rural HBT regions of northern Bangladesh have a somewhat intermediate nutritional condition. In India found that stunting was more in the younger age group than older among the tribal adolescent girls [33], the result supported to our study finding.

According to studies on school-aged children in other districts of Bangladesh, 38.0% of children in Kishoreganj were stunting, while 49.0% and 32.0% of children in Sylhet were thinness and stunting, respectively [34, 35]. This suggests that school-aged children in rural HBT region have better nutritional status than other locations. Studies have shown regional, socioeconomic and ethnic variations in the nutritional status of school-age children in neighbouring India as well. Among primary school children in Maharashtra (Western India), stunting was 19.4% [36]; In Kolkata (Eastern India), 23.6% thinness and 10.6% stunting were found among slum-dwelling school-age children [37], 17.9% stunting among Santal tribal children (aged 5–12 years) in Purulia of West Bengal (Eastern India) [38]; In Varanasi (Northern region), 27% of school-age children were stunted [39]. This study found a coexistence of food insecurity and child undernutrition, with similar findings observed in Ethiopia and Nigeria indicating that food insecurity increases the prevalence of thinness among school-going children [11, 27]. According to another study conducted in Bangladesh, among school-going children of tea plantation workers had a higher prevalence of food insecurity, which was associated with stunting and thinness [35]. The UNICEF has highlighted household food insecurity, inadequate child care and unhealthy household environment and a lack of health services as the major causes of malnutrition in children [40].

### Strength and limitation of this study

The present study investigated the nutritional status and its determinants among T and NT schoolchildren using primary data in Bangladesh’s HBT area. However, the study had certain shortcomings. First, we could not further monitor the situation of children nutritional status, because we conducted cross-sectional study. Moreover, this study was confined to a specific rural geographical area, so it was not possible to generalize the results for all Bangladeshi schoolchildren. Our sample included a reasonably small number of T children because they form small proportion of children in the study area. We had to use a combination of probability and non-probability sampling methods due to logistical constraints that prevented us from using probability sampling exclusively.

Based on the limitations identified, this study could be extended by conducting further studies may consider monitoring nutritional studies of T children in a longitudinal investigation focusing on all factors and including children, adolescents and young adults.

## Conclusions

The study focused on children between the ages of 6 and 13 in the HBT region of the Rajshahi district, revealing that both tribal and non-tribal children were suffering from undernutrition, such as thinness and stunting. Although the prevalence of these issues was marginally lower among tribal children compared to non-tribal children, this disparity was not statistically significant. The research also compared its findings to similar studies conducted nationally and internationally and discovered that the prevalence of thinness and stunting among tribal and non-tribal communities was similar or lower. According to the WHO standard z-scores, it was evident that both tribal and non-tribal children exhibited signs of undernutrition in their overall nutritional status. Several factors were noted to be significantly linked to thinness and stunting, including the poor nutritional status of parents, household food insecurity, and the physical activities of the children, such as regular outdoor play and frequent TV watching. Notably, household food insecurity emerged as the most critical factor associated with undernutrition in both T and NT children.

### Recommendations and policy implications

Based on our findings, the Government of Bangladesh should attempt to ameliorate the problem of school-going children and parents’ malnutrition. Special focus should be given to the modifiable factors associated with malnutrition. In order to enhance the nutritional status of T and poor household children, there is an urgent need to increase accessibility to the social safety net and give stipend to T and poor school children. Ensure and strengthen school-based health promotion activities of the Government, such as deworming, vitamin A capsule distribution, and periodic health examinations of the children. It is also needed to make awareness and mobilization of tribal and poor household parents about their child’s nutritional status.

## Data Availability

This study was based on the primary data. The data are available from the corresponding author on reasonable request.

## Abbreviations

aOR: Adjusted Odds Ratio
BAZ: BMI-for-age z-score
BDT: Bangladesh Taka
BMI: Body mass index
CI: Confidence interval
cOR: Crude Odds Ratio
HAZ: Height-for-age z-score
HBT: High Barind Tract
LMIC: Low- and middle-income countries
MD: Mean difference
NT: Non-tribal
SD: Standard Deviation
SDGs: Sustainable Development Goals
SPSS: Statistical package for the social sciences
T: Tribal
TV: Television
UNICEF: United Nations Children’s Fund
VIF: Variance Inflation Factor
WHO: World Health Organization
