# Correction: Nutritional status of tribal and non-tribal school-going children in rural Bangladesh: A comparative study

**DOI:** 10.1186/s12889-024-20792-3

**Published:** 2024-11-21

**Authors:** Reazul Karim, Ramendra Nath Kundu, Sifat Hossain, Susmita Bharati, Premananda Bharati, Golam Hossain

**Affiliations:** 1 Health Research Group, Department of Statistics, University of Rajshahi, Rajshahi, 6205 Bangladesh; 2 Department of Anthropology, (UGC-NET), West Bengal State University, West Bengal, India; 3 Sociological Research Unit, Indian Statistical Institute, Kolkata, West Bengal India; 4 Former Professor and Head, Biological Anthropology Unit, Indian Statistical Institute, Kolkata, West Bengal India

**BMC Public Health (2024) 24:2975**

10.1186/s12889-024-20487-9

The original publication of this article contained an incorrect corresponding author. The correct corresponding author is shown on this correction article, the original article has been updated.

